# Reactive Oxygen Species and Their Involvement in Red Blood Cell Damage in Chronic Kidney Disease

**DOI:** 10.1155/2021/6639199

**Published:** 2021-02-25

**Authors:** Krzysztof Gwozdzinski, Anna Pieniazek, Lukasz Gwozdzinski

**Affiliations:** ^1^Department of Molecular Biophysics, Faculty of Biology and Environmental Protection, University of Lodz, Lodz, Poland; ^2^Department of Pharmacology and Toxicology, Medical University of Lodz, Lodz, Poland

## Abstract

Reactive oxygen species (ROS) released in cells are signaling molecules but can also modify signaling proteins. Red blood cells perform a major role in maintaining the balance of the redox in the blood. The main cytosolic protein of RBC is hemoglobin (Hb), which accounts for 95-97%. Most other proteins are involved in protecting the blood cell from oxidative stress. Hemoglobin is a major factor in initiating oxidative stress within the erythrocyte. RBCs can also be damaged by exogenous oxidants. Hb autoxidation leads to the generation of a superoxide radical, of which the catalyzed or spontaneous dismutation produces hydrogen peroxide. Both oxidants induce hemichrome formation, heme degradation, and release of free iron which is a catalyst for free radical reactions. To maintain the redox balance, appropriate antioxidants are present in the cytosol, such as superoxide dismutase (SOD), catalase (CAT), glutathione peroxidase (GPx), and peroxiredoxin 2 (PRDX2), as well as low molecular weight antioxidants: glutathione, ascorbic acid, lipoic acid, *α*-tocopherol, *β*-carotene, and others. Redox imbalance leads to oxidative stress and may be associated with overproduction of ROS and/or insufficient capacity of the antioxidant system. Oxidative stress performs a key role in CKD as evidenced by the high level of markers associated with oxidative damage to proteins, lipids, and DNA *in vivo*. In addition to the overproduction of ROS, a reduced antioxidant capacity is observed, associated with a decrease in the activity of SOD, GPx, PRDX2, and low molecular weight antioxidants. In addition, hemodialysis is accompanied by oxidative stress in which low-biocompatibility dialysis membranes activate phagocytic cells, especially neutrophils and monocytes, leading to a respiratory burst. This review shows the production of ROS under normal conditions and CKD and its impact on disease progression. Oxidative damage to red blood cells (RBCs) in CKD and their contribution to cardiovascular disease are also discussed.

## 1. Introduction

Chronic kidney disease (CKD) is a pathological condition in which, as a result of impaired excretory function, associated with a decrease in the number of nephrons, toxic substances accumulate in the body. Waste products that are normally excreted by the kidneys in the urine accumulate in amounts that are toxic to the body and are referred to as uremic toxins. Many of the uremic toxins retained in the body exhibit biological/biochemical activity contributing to the development of the uremic syndrome and endogenous poisoning of the body [1]. Many of them cause chronic inflammation and oxidative stress. Both inflammation and oxidative stress can contribute to the development of chronic kidney disease and its many complications. The release of ROS in the body is related to their physiological role as signaling molecules. However, their increased production and/or insufficient performance of antioxidant systems can lead to oxidative stress which is associated with the damage or/and oxidative modification of vital molecules such as nucleic acid, proteins (enzymes), and lipids [2].

In chronic kidney disease, oxidative stress performs a key role in disease progression as evidenced by the high level of markers associated with oxidative damage to proteins, lipids, and DNA *in vivo*. Oxidative stress is related to the overproduction of reactive oxygen species (ROS) and reduced antioxidant capacity in which there is a decrease in the activity of SOD, glutathione peroxidase, peroxiredoxin 2, and antioxidants with low molecular weight, such as glutathione and vitamins C, A, and E. An additional factor increasing oxidative stress is low-biocompatibility dialysis membrane treatments used in hemodialysis, which lead to the activation of phagocytic cells, especially neutrophils and monocytes [3]. This review shows the production of ROS in normal conditions and in chronic kidney disease and their impact on disease development.

Maintaining the balance between ROS production and utilization has an important role in cell signaling and hemostasis. This balance is also important for the functioning of blood vessels. Its disturbance related to the excessive production of ROS due to acute infection or inflammation may lead to the damage of biological material [4]. ROS is involved in the pathogenesis of many diseases, including cardiovascular diseases such as hypertension, ischemic heart disease, and thrombosis [5].

In this review, the oxidative damage of red blood cells in CKD, which affects the rheological properties of the blood but is also associated with the development of cardiovascular diseases, was also taken into consideration. We also emphasize the role of oxidative stress, which disrupts redox homeostasis, exacerbates the disease in patients with chronic kidney disease, and is a major contributor to the cardiovascular disease that accompanies chronic kidney disease.

## 2. ROS Production and Oxidative Stress

Oxidative stress is a consequence of living under aerobic conditions. ROS are released in organisms under normal physiological conditions and act as signal molecules. However, their overproduction and/or antioxidant system failure can lead to oxidative stress. RFT as strong oxidizing agents lead to the damage/modification of life-important molecules such as DNA, proteins, and lipids. The precursor of ROS is the superoxide anion radical (O_2_^•-^), which is the product of one-electron reduction of molecular oxygen. Superoxide is usually formed in the body by catalyzed reactions and/or as a result of nonenzymatic electron transfer, when the electron is converted to molecular oxygen [6]. The main source of reactive oxygen species is mitochondria, where there is a leakage of electrons in the respiratory chain and reduction of molecular oxygen. There are 11 sites in the mitochondria that generate the superoxide radical [7]. Large amounts of superoxide are produced by NADPH oxidase (NOX) from the cytoplasmic membrane and from the enzyme complex of the mitochondrial electron transport chain, but also from sources such as xanthine oxidase (XO), lipoxygenase (LOX), cyclooxygenase (COX), and cytochrome P450 (endoplasmic reticulum) from peroxisomes of other organelles [8–10]. Other sources of ROS are the endoplasmic reticulum, nuclear envelope, cytoplasm, and endosomal and plasma membranes [9, 10]. ROS are also produced by cytoplasmic membranes, lysosomes, mitochondria, and peroxisomes [11, 12]. NADPH oxidase catalyzes the one-electron reduction of molecular oxygen, thus producing O_2_^•-^. Supposedly 1% to 3% of oxygen running through the mitochondria is reduced to O_2_^•-^ [13].

Xanthine oxidase which is an important enzyme that contributes significantly to the production of superoxide in ischemia-reperfusion can also reduce nitrite to nitric oxide and may be a potential source of peroxynitrite (ONOO^−^) [14]. Generally, superoxide, which is a precursor to other reactive oxygen species, shows low reactivity with few exceptions. One of them is the reaction with nitric oxide, and the other is the Haber-Weiss reaction catalyzed by transition metal ions (Fe, Cu, Ti, Ni, etc.) and the spontaneous dismutation reaction (O_2_^•-^/HO_2_^•^) and by superoxide dismutase with the constant rate of 10^5^ to 10^9^ mol^−1^ × s^−1^ at pH 7, respectively [15]. However, this reactivity increases with its protonated form, i.e., hydroperoxide radical (perhydroxyl radical), which can initiate lipid peroxidation and thiol oxidation [16].

Disproportionation reaction of superoxide leads to hydrogen peroxide, which is a signaling molecule but also a strong oxidant. However, it does not react with most biological molecules due to its high activation energy barrier, but it can oxidize thiols [17]. An important reaction, also *in vivo*, is its reduction catalyzed by transition metals and superoxide. To maintain the conditions of proper homeostasis, cells have at their disposal antioxidant enzymes such as superoxide dismutase [18], catalase [19], glutathione peroxidases [20], heme oxygenase-1 (HO-1) [21], the thioredoxin system [22], and low molecular weight antioxidants soluble in water (glutathione [23], ascorbic acid) and soluble in lipid (*α*-tocopherol, ubiquinol, and *β*-carotene) [24]. Despite the well-developed antioxidant system, vital particles and macromolecules are damaged. The Fenton reaction in which hydrogen peroxide is reduced to a hydroxyl radical by Fe(II) is of key importance here. The ^•^OH radical is the most reactive form of oxygen and is one of the strongest oxidants. Most reactions of the ^•^OH radical with biological molecules, such as proteins (e.g., albumin and hemoglobin), aromatic amino acids, unsaturated fatty acids, DNA bases, or ascorbic acid, occur with constant rates of >10^10^ (mol^−1^ × s^−1^) and are diffusion-controlled reactions [15]. Hydroxyl radical has a very short lifetime, and its radius of action is 10^−8^ m [15]. Interestingly, Fe(II) ions on the water surface react with H_2_O_2_ more than 100 times faster than those in water [25]. Another radical is nitric oxide released by most of our body's cells, and it is a vasodilator and, therefore, leads to lowering of blood pressure and increased blood flow. NO^•^ is synthesized from L-arginine by oxidation of the guanidine group in the presence of stereospecific enzyme NO^•^ synthase and NADPH and tetrahydrobiopterin as cofactors [26].

Hemoglobin is not only a protein that supplies nonoxygen to tissues but also a nitric oxide transporter. By supplying NO^•^, it regulates the tension of blood vessels. The autooxidation of hemoglobin causes the formation of methemoglobin (MetHb), which leads to inflammation associated with the release of heme from MetHb. In normally functioning erythrocytes, the redox state is maintained due to the presence of methemoglobin reductase. This enzyme with the participation of NADPH reduces Fe(III) in MetHb to Fe(II) present in Hb. Nitric oxide is released into the lumen of the vessel and is captured by red blood cells (RBCs) [27, 28]. Inside, the NO^•^ is bound by a hemoglobin molecule to form S-nitrosohemoglobin (HbFe(II)SNO) [27, 29, 30]. Under anaerobic conditions, hemoglobin (deoxyhemoglobin) may bind to nitric oxide to form nitrosyl-hemoglobin (HbFe(II)NO) (reaction (1)):

(1) $$\begin{array}{c} \text{Hb Fe }\left(\text{II}\right)+{\text{NO}}^{\cdot }\to \text{Hb Fe }\left(\text{II}\right)\text{NO} \end{array}$$

The concentration of HbFe(II)NO in venous blood is approx. 30-fold higher than that of S-nitrosohemoglobin, while in arterial blood, it is only approx. 2-fold [31]. However, the reaction of oxyHb with NO^•^ leads to oxidation of oxyHb to metHb and nitrate [32]. This reaction (reaction (2)) is irreversible and causes a decrease in the bioavailability of nitric oxide, thereby interfering with the vasodilator dependent on the blood vessels [33].

(2) $$\begin{array}{c} \text{Hb Fe }\left(\text{II}\right){\text{O}}_{2}+{\text{NO}}^{\cdot }\to \text{Hb Fe }\left(\text{III}\right){\text{NO}}_{3^{-}} \end{array}$$

This mechanism is crucial in the expansion of blood vessels with the participation of NO^•^. Even a small degree of hemolysis can lead to NO^•^ binding and inhibit endothelium-dependent vasodilation [32]. In turn, oxyHb released in plasma can react with NO^•^ and produce ONOO^−^/ONOOH and metHb [34]. It was shown that the treatment of red blood cells with nitric oxide led to metHb formation and oxidative damage of lipids and proteins in these cells [35]. An important group of compounds are quinones, of which the reduction leads to the formation of reactive semiquinones (Q^•-^). An example would be reduction by xanthine oxidase in a nitrogen atmosphere, which is a method that ensures a continuous production of semiquinone. Semiquinones can also be formed by the autooxidation of hydroquinones. Semiquinones may react with hydrogen peroxide generating the hydroxyl radical (reaction (3)) [36]:

(3) $$\begin{array}{c} {\text{Q}}^{\cdot -}+{\text{H}}_{2}{\text{O}}_{2}\to \text{Q}+\text{H}{\text{O}}^{\cdot }+\text{H}{\text{O}}^{-} \end{array}$$

Some xenobiotics and drugs, e.g., adriamycin (doxorubicin) with xanthine oxidase and xanthine in an oxygen-free atmosphere in the presence of H_2_O_2_, resulted in the production of hydroxyl radicals in a similar manner [37]. Similar to other heme-containing proteins such as cytochrome c (associated with electron transport) and catalase or cytochrome oxidase, which are proteins involved in the breakdown of peroxides, Hb and Mb may also exhibit similar properties to other heme proteins. As a result of the oxidation of both proteins, toxic derivatives are formed, such as ferryl forms, ferrylmyoglobin Mb(FeIV=O), and ferrylhemoglobin Hb(FeIV=O), respectively, as well as radical ferryl forms, formed as a result of the oxidation of metmyoglobin and methemoglobin: Mb(FeIV=O‧‧‧Tyr^•^) and Hb(FeIV=O‧‧‧Tyr^•^), respectively, with the location of the unpaired electron on the rest of Tyr(*β*145) of the globin chain [38, 39]. It has been shown that both hemoproteins Hb and Mb in an oxidized state, for example, in ferryl and ferryl radical forms, can induce lipid peroxidation by abstraction of a hydrogen atom in the hydrocarbon chain [39, 40]. Relations (4) and (5) show ferryl and ferryl radical formation from the porphyrin (Por) compound including myoglobin and hemoglobin.

(4) $$\begin{array}{c} \text{Por}‐\text{Fe}\left(\text{II}\right)+{\text{H}}_{2}{\text{O}}_{2}\to \text{Por}‐\text{Fe}\left(\text{IV}\right)=\text{O}+{\text{H}}_{2}\text{O} \end{array}$$

(5) $$\begin{array}{c} \text{Por}‐\text{Fe}\left(\text{III}\right)+{\text{H}}_{2}{\text{O}}_{2}\to {\text{Por}}^{\cdot }+\text{Fe}\left(\text{IV}\right)=\text{O}+{\text{H}}_{2}\text{O} \end{array}$$

Ferryl forms can be reduced by myoglobin or hemoglobin to metMb and metHb, respectively (reaction (6)).

(6) $$\begin{array}{c} \text{Por}‐\text{Fe}\left(\text{IV}\right)=\text{O}+\text{Por}‐\text{Fe}\left(\text{II}\right)+2{\text{H}}^{+}\to 2\text{Por}‐\text{Fe}\left(\text{III}\right)+{\text{H}}_{2}\text{O} \end{array}$$

The ferryl form and the ferryl radical form were first discovered in horseradish peroxidase, but it is now known that these forms are found throughout the heme enzyme family, which includes all peroxidases, heme catalases, P450, cytochrome oxidase, and NO synthase [41]. The ferryl form of myoglobin initiated the process of lipid peroxidation in the membranes to form isoprostane, as well as the reduction of ascorbates or urates [39].

In inflammation, neutrophil accumulation occurs, which as a result of activation, in addition to superoxide and hydrogen peroxide, produces hypochlorous acid (HClO), which is produced in the oxidation reaction of chlorides by hydrogen peroxide catalyzed by myeloperoxidase (MPO) [42]. HClO is a strong oxidant capable of oxidative modification of molecules and macromolecules. Hypochlorous acid shows a strong affinity for low molecular weight thiols and protein thiols but also to methionine. It leads to the oxidation of glutathione (GSH) to oxidized glutathione (GSSG) [43]. HClO causes chlorination of tyrosine to form two derivatives 3-chlorotyrosine and 3,5-dichlorotyrosine in proteins and peptides [44]. HClO reacts with amino groups to form chloramines. In reaction with proteins and peptides, carbonyl compounds are formed [45]. Another way of forming aldehydes is through the breakdown of chloramines. In addition, hypochlorous acid reacts with compounds that contain a double bond to form chlorohydrins. In the case of biological material, it reacts with unsaturated fatty acids and cholesterol to form the corresponding chlorohydrins [43]. The reaction of hypochlorous acid with reducing agents such as Fe(II) and superoxide, which is another source of the hydroxyl radical, is also important (reactions (7) and (8)) [46]:

(7) $$\begin{array}{c} \text{Fe}\left(\text{II}\right)+\text{HC}1\text{O}\to \text{Fe}\left(\text{III}\right)+\text{H}{\text{O}}^{\cdot }+\text{C}1^{-} \end{array}$$

(8) $$\begin{array}{c} {{\text{O}}_{2}}^{\cdot -}+\text{HC}1\text{O}\to {\text{O}}_{2}+\text{H}{\text{O}}^{\cdot }+\text{C}1^{-} \end{array}$$

Myeloperoxidase (MPO) can also directly convert superoxide to singlet oxygen (^1^O_2_). ^1^O_2_ can also be produced in a reaction of HClO with H_2_O_2_ [47]. Singlet oxygen is also produced in many enzymatic reactions in which heme proteins, lipoxygenases, and activated leukocytes participate, as well as in nonenzymatic reactions involving free radicals. ^1^O_2_ is involved in the oxidation of proteins, leading to changes in both the side chains and the main backbone of amino acids, peptides, and proteins. It also forms reactive peroxides with Tyr, His, and Trp residues, which may further be involved in protein oxidation [48].

## 3. Protection of Cells and Tissues against Oxidative Stress

The role of antioxidants is to inactivate ROS which initiate oxidative damage. The imbalance between oxidants and antioxidant systems causes oxidative damage in the cell, which leads to overexpression of oncogene genes, generation of mutagenic compounds, and promotion of atherosclerotic activity and in consequence to cancer, neurodegenerative diseases, cardiovascular diseases, diabetes, and kidney diseases.

Antioxidants act to directly scavenge oxygen free radicals, and other oxidizing molecules, and regenerate damaged biomolecules. Typically, antioxidants are classified into two groups. The first line of defense includes antioxidant enzymes, which include superoxide dismutase, catalase, and glutathione peroxidase. The second group of nonenzymatic antioxidant consists of low molecular weight antioxidants that can be divided into antioxidants soluble in the water environment and in the lipid environment [49, 50]. The additional group consists of repair systems that regenerate oxidatively damaged biopolymers, remove oxidized proteins by proteolytic enzymes, and repair oxidized lipids with the participation of phospholipases, peroxidases, or acyl transferases [51, 52]. Another group is represented by enzymatic systems that repair nucleic acids damaged by oxidation [53].

Primary antioxidants react directly with free radicals (hydroxyl HO^•^, alkoxyl RO^•^/lipoxyl LO^•^, or peroxyl ROO^•^) through the donation of a hydrogen atom, interrupting chain reactions. Secondary antioxidants include, for example, singlet oxygen quenchers, metal chelators, and inhibitors of oxidizing enzymes such as COX, LOX, and NADH oxidase [50].

The maintenance of redox homeostasis involves antioxidant enzymes such as superoxide dismutase, catalase, glutathione peroxidase, glutathione reductase, and nonenzymatic systems such as proteins (ferritin, transferrin, ceruloplasmin, and albumin) and low molecular weight antioxidants, like glutathione, ascorbic acid, uric acid, coenzyme Q, and lipoic acid [52].

The superoxide anion radical, a precursor to other ROS, is transformed by superoxide dismutase to H_2_O_2_. In mammals, there are three types of superoxide dismutase: zinc-copper dismutase (SOD1) which is found in the cytosol, manganese superoxide dismutase (SOD2) which is found in the mitochondria, and extracellular superoxide dismutase (SOD3).

The hydrogen peroxide produced during dismutation of superoxide is reduced to water by catalase. This enzyme is present in most cells, organs, and tissues and at elevated concentrations in the liver and erythrocytes [54]. Other enzymes that remove hydrogen peroxide are peroxiredoxin (Prx), thioredoxin reductase (TrxR), and glutathione peroxidase (GPx) [55]. GPx, in addition to the decomposition of H_2_O_2_, also breaks down organic peroxides into alcohols and oxygen. A similar function is also performed by glutathione S-transferases (GST), which can reduce lipid hydroperoxides. Thioredoxins (Trxs) and glutaredoxins (Grxs) repair oxidized cysteine residues. Thioredoxin reductase catalyzes the reduction of the disulfide at the Trx active site [56]. TrxR also participates in the regeneration of other antioxidant molecules, such as dehydroascorbate, lipoic acid, and ubiquinone [57].

The “second line of defense” consists mainly of reduced thiols and low molecular weight (LMW) antioxidants, both water- and fat-soluble, reduced glutathione, ascorbate, tocopherols, retinols, and others. LMW antioxidant can move to specific places in cells in which oxidative damage occurs [58, 59].

Another important group of antioxidants is thiols, which react with most of the physiological oxidants. They are important in maintaining the homeostatic intracellular and tissue redox status based on the redox pair. Multiple studies show that the redox state in cells is important for ROS-mediated signaling and mitochondrial function [60]. Thiols are sensitive to oxidation which leads to the formation of dithiol/disulfide. This reaction occurs in the case of glutathione, thioredoxins (with -SH groups in the active center), and other proteins containing cysteine [61]. Glutathione (GSH) is one of the most important intracellular antioxidants because its concentration is high and ranges from 5 to 10 mM. Multiple studies show that the redox state in cells is important for ROS-mediated signaling and mitochondrial function [60]. The decrease in GSH concentration in the cytosol leads to an increase in the production of mitochondrial ROS and depolarization of the mitochondrial membrane [62]. Glutathione, as a water-soluble antioxidant, primarily protects the proteins present in the cytosol. As an antioxidant, it reacts with O_2_^•-^ and HO^•^ radicals, hydrogen peroxide, and chlorinated oxidants [61].

Cysteine-rich proteins and peptides can bind to heavy metals due to the presence of thiol groups. A special group here is metallothioneins (MT), peptides, and proteins with a molecular mass ranging from 500 to 14000 g/mol located in the membrane of the Golgi apparatus, which bind to both physiological metals such as zinc, copper, and selenium and toxic heavy metals including cadmium, mercury, silver, lead, arsenic, manganese, cobalt, and nickel. MT regulate zinc levels and the distribution in the intracellular space. In addition to zinc-metallothionein interactions, MT is an important regulator of glutathione synthesis [63]. In the Zn-MT complex, a cysteine residue may induce redox properties to participate in the MT redox cycle. Moreover, MT has an antioxidant effect, taking part in the inactivation of reactive oxygen and nitrogen species, including free radicals, which has been proven in many *in vivo* and *in vitro* studies [64, 65].

**Figure 1:**
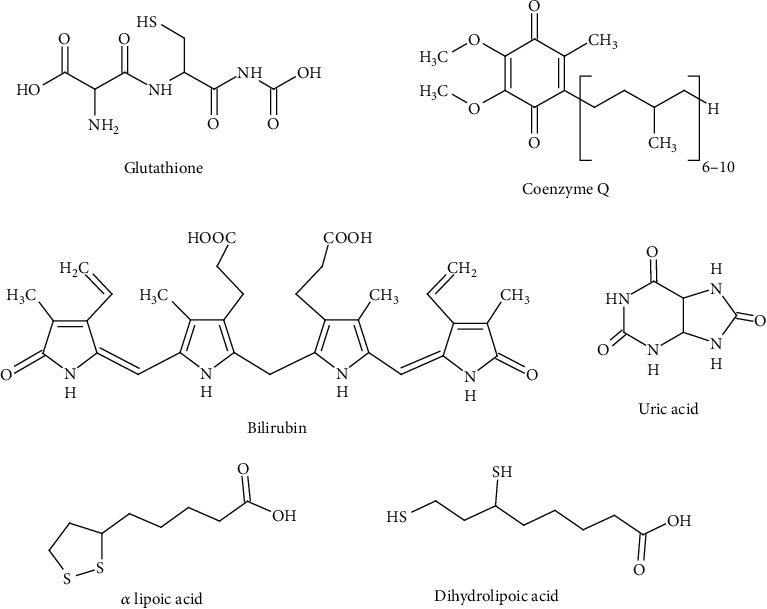
Endogenous low molecular weight antioxidants.

Ascorbic acid/ascorbate (vitamin C), soluble in water, is an important and ubiquitous antioxidant that is easily oxidized to dehydroascorbic acid (Figure 1). Ascorbic acid assists in the maintenance of the integrity of blood vessels and connective tissue, takes part in iron absorption, and participates in neuroprotection and hematopoiesis [66, 67]. It also protects membrane lipids from peroxidation and is an important antioxidant that protects the brain tissue and is involved in the biosynthesis of catecholamines [68]. Ascorbic acid protects membranes and other hydrophobic compartments from oxidative damage by regenerating the antioxidant form of vitamin E. In addition, ascorbic acid effectively reacts directly with HO^•^ radicals and peroxide radicals with rate constants from 10^6^ to 10^8^ M^−1^ s^-l^. It is also a singlet oxygen scavenger [69]. Although ascorbic acid does not directly remove lipophilic radicals, it acts synergistically in conjunction with tocopherol to remove lipid peroxide radicals. Moreover, it reacts with the membrane-bound tocopheroxyl radical regenerating it to the active tocopherol [70].

Another antioxidant with a low molecular weight is the fat-soluble *α*-tocopherol (vitamin E) containing in the structure a chroman ring with a branched saturated side chain (Figure 1). Natural vitamin E consists of *α*-, *β*-, and *γ*-tocopherols, but the greatest share is held by *α*-tocopherol. Vitamin E protects lipids against peroxidation by interrupting free radical chain reactions by providing a hydrogen atom with reactive lipid (L^•^), lipoxyl (LO^•^), and peroxyl (LOO^•^) radicals, forming lipids, alcohols, and hydroperoxides, respectively. The resulting tocopheryloxyl radical is regenerated by ascorbic acid and/or glutathione [71]. Vitamin E also protects low-density lipoproteins against free radical damage. *α*-Tocopherol inhibits proatherogenic processes through the proliferation of smooth muscle cells in vivo and adhesion of monocytes to the endothelium [72]. *α*-Tocopherol also contributes to the stabilization of atherosclerosis [72, 73]. Vitamin E performs a protective role in the formation of cancer, the aging process, arthritis, and cataracts. It can also prevent excessive platelet aggregation that can lead to atherosclerosis; in addition, it also helps to reduce the production of prostaglandins such as thromboxane, which cause platelets to stick together [74].

*β*-Carotene (*β*-Car) is an antioxidant that is soluble in the lipid environment. It belongs to the carotenes, which are terpenoids (isoprenoids) (Figure 1). *β*-Carotene, unlike lycopene, has beta-cyclohexene rings at both ends of the molecule. *β*-Car is a highly effective physical singlet oxygen quencher, which is formed in the skin with the help of endogenous photosensitizers under the influence of sunlight [75]. It also participates in quenching singlet oxygen, which contributes to cataract formation and macular degeneration in the eye [76]. The action of *β*-Car is here supported by another carotenoid, lycopene, and *α*-tocopherol. The antioxidant activity of *β*-carotene was comparable to that of *α*-tocopherol [77].

Coenzyme Q10 (CoQ, ubiquinone) is a benzoquinone with 10 isoprenyl units in its fat-soluble side chain (Figure 1). CoQ is crucial in the mitochondrial electron transport chain [78]. It occurs especially in the heart, skeletal muscle, liver, kidney, and brain [79]. Its low concentration in plasma can lead to cardiovascular disorders. Coenzyme Q10 is an intracellular antioxidant, but it is also present in plasma to protect LDL lipoproteins and cell membranes from oxidative damage [80]. Coenzyme Q10 reduces the oxidized form of vitamin E, restoring its antioxidant properties [69]. The reduced form of coenzyme Q10 inactivates carbon-centered lipid radicals and lipid peroxyl radicals [81]. On the other hand, CoQ may exhibit prooxidative properties, as its single-electron reduction leads to a semiquinone which, when reacting with hydrogen peroxide, generates a highly reactive hydroxyl radical [82].

*α*-Lipoic acid (LA) is a short-chain fatty acid containing a five-membered ring (dithiolane ring) with two sulfur atoms. Lipoic acid is unique in its solubility as it is soluble in both water and lipids. However, its reduced form contains two groups -SH (dihydrolipoic acid (DHLA)) (Figure 1). It has been shown that both the oxidized and reduced forms have antioxidant properties in the inactivation of free radicals and other reactive oxygen species such as hydrogen peroxide and hypochlorous acid, as well as the ability to chelate transition metals [83, 84]. LA and DHLA effectively chelate directly toxic metals such as manganese, zinc, cadmium, lead, cobalt, nickel, iron, copper, cadmium, arsenic, and mercury. Moreover, they show the properties of regeneration of endogenous antioxidants such as glutathione, vitamin C, and vitamin E, metal chelating activity, and repair of oxidized proteins [85]. Lipoic acid and dihydrolipoic acid are involved in the prevention of cardiovascular diseases, and they also have anti-inflammatory, anticancer, antiaging, and neuroprotective properties [86].

One of the low molecular weight antioxidants is uric acid (UA), present in plasma. Uric acid inactivates the hydroxyl and peroxyl radicals and is an effective singlet oxygen scavenger (Figure 1). It has been shown to protect the erythrocyte membrane against lipid peroxidation. It has also been reported that uric acid is a unique scavenger of peroxynitrite in the extracellular space [87]. In experimental allergic encephalomyelitis (EAE), uric acid inhibited the nitration of neuronal proteins via peroxynitrite and inhibited the growth of the blood-brain barrier, resulting in less leukocyte infiltration [88]. However, the protective effect of UA may not be related to direct inactivation of peroxynitrite in neurons but may be due to a reduction in endothelial nitric oxide levels. Uric acid has also been shown to reduce the bioavailability of nitric oxide in endothelial cells [89]. On the other hand, the prooxidative effect of UA, which appears in cardiovascular diseases and may perform a role in the pathogenesis of these diseases, is also shown [90].

Bilirubin (BIL) belongs to amphiphilic antioxidants and has effective cytoprotective activity in relation to lipids (Figure 1). Acting as an antioxidant, it is oxidized to biliverdin. In turn, biliverdin is reduced to bilirubin by biliverdin reductase. BIL inactivates the hydroxyl, superoxide anion, and nitric oxide radicals and shows excellent protective activity against mitochondrial oxidative stress [91]. However, the nanomolar concentrations of bilirubin in tissues (about 20-50 nM) are far too low to counteract the activities of the reactive oxygen species found in millimolar concentrations [92].

The nonenzymatic antioxidants also include metal-binding proteins, which include transferrin, ferritin, lactoferrin, and ceruloplasmin. Their mechanism of action is related to the sequestration of transition metal ions that catalyze the reactions in which most of the oxygen-derivative radicals are formed, including the Fenton and Haber-Weiss reactions. Transferrin is the main iron-binding protein in the blood. Its low iron saturation, about 15%, indicates anemia, while high saturation, over 60%, indicates iron overload or hemochromatosis [93]. Another iron-binding protein is ferritin, found in cells in the cytosol, but small amounts are found in plasma where it acts as a carrier for iron. The protein not only binds to iron but also releases it in a controlled manner. Plasma ferritin is also a marker of the total amount of iron stored in the body [94]. Lactoferrin found in the milk of mammals is a protein that is also found in saliva, tears, and nasal secretions. This protein binds to iron and is carried through various receptors to and between cells, serum, bile, and cerebrospinal fluid. Lactoferrin is one of the components of the body's immune system, is part of the innate defense, and has antibacterial and antiviral properties [95].

Ceruloplasmin is an enzymatic glycoprotein containing 6 copper ions. It is the major copper-carrying protein in the blood. Ceruloplasmin has a ferroxidase activity that is important in iron homeostasis and defense mechanisms in oxidative stress. Its main role is related to the oxidation of Fe(II) to Fe(III), which in the oxidized form can be transported by transferrin and bind to ferritin [96]. Certain mutations in the ceruloplasmin gene lead to disturbances in iron metabolism and distribution, leading to massive Fe accumulation in the liver, brain, and pancreas, as well as problems with the retina and diabetes [96]. Ceruloplasmin is an important antioxidant that protects biomolecules from damage induced by free oxygen radicals. Ceruloplasmin was shown to be a much more effective scavenger of peroxide radicals than SOD, deferoxamine, and albumin, but slightly less effective than catalase [97]. Ceruloplasmin, regardless of its catalytic activity of peroxidase, is an effective antioxidant that breaks the chains of free radical reactions.

**Figure 2:**
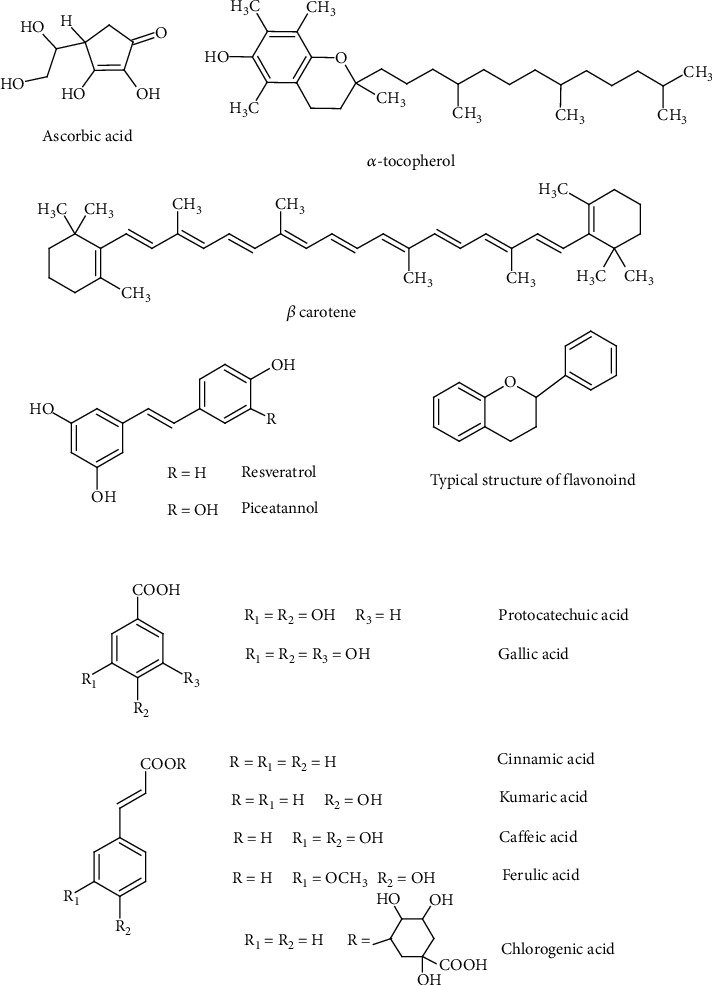
Exogenous low molecular weight antioxidants.

In addition to endogenous antioxidants such as GSH, UA, BIL, CoQ, LA, DHLA, and polyamines such as spermine, spermidine, and putrescine [98], the remaining antioxidants enter the body through food, mainly from vegetables and fruits. Antioxidant properties characterize also monophenol, diphenol, and polyphenol derivatives. The monophenols include derivatives of benzoic and cinnamic acid and their esters, most often methyl, propyl, and lauryl (Figure 2). Derivatives of benzoic acid include protocatechuic acid and gallic acid, and derivatives of cinnamic acid include coumaric acid, caffeic acid, ferulic acid, and chlorogenic acid effective free radical scavengers [99, 100]. The next group is diphenol derivatives (stilbene derivatives), resveratrol, and picetannol (Figure 2) [101]. Flavonoids are a large group of compounds with antioxidant properties, inactivating free radicals and other ROS, inhibiting prooxidative enzymes such as cyclooxygenases, lipoxygenases, and oxidase, chelating heavy metals, and modulating antioxidant enzymes [102, 103]. In addition, the flavonoids have antioxidant, immunomodulatory, anti-inflammatory, and anticancer properties, as well as potential antiviral effects [104, 105]. Currently, there are over 8,000 flavonoids known, and their structure is based on the chroman ring with a phenyl substituent, which is present in the 2, 3, or 4 positions (Figure 2). Depending on the position of the phenyl substituent, we have flavonoids, isoflavonoids, or neoflavonoids. Their antioxidant properties are determined by the number of hydroxyl groups present in the phenyl substituent and associated with the chroman ring, as well as the presence of a carbonyl group and a double bond in the ring. Additionally, hydroxyl groups can be ester bound to organic acids such as gallic, malonic, malic, ferulic, and others or/and form O- or C-glycosidic bonds with sugar residues [104, 106]. Flavonoids can condense to form tannins, oligomers, dimers to pentamers, and sometimes polymers composed of 14-15 monomer molecules [107]. The introduction of two double bonds into the chroman ring leads to the formation of anthocyanins and anthocyanidins of colored pigments, characterized by the presence of a positive charge (flavylium ion) [103].

## 4. Oxidative Stress in CKD Patients

Permanent oxidative stress occurs in patients with chronic kidney disease. Markers of oxidative stress associated with the progression of CKD can be measured in body fluids such as plasma, red blood cells, and urine (Table 1). In plasma and saliva, antioxidant enzymes (SOD, Cat, GPx, and Trx) and low molecular weight antioxidants (GSH, Vit. C and E, and *β*-carotene), protein oxidation products (SOPPs, AGEs, and protein carbonyls), and lipids (MDA, 4-HNE, and F2 isoprostanes) are determined, while in the urine, the oxidation products of nucleic acids 8-OHG (8-hydroxyguanosine) and 8-OHdG (8-hydroxy-2′-deoxyguanosine) are determined [108, 109]. Another proposed biomarker that can be measured in the urine is neutrophil gelatinase-associated lipocalin (NGAL) resistant to degradation and rapidly excreted in the urine. NGAL is now an approved biomarker of CKD progression [110, 111].

Less commonly used markers include thiols and oxLDL. It has been observed that patients with chronic CKD have increased levels of plasma thiol oxidation even contributing to progressive renal dysfunction [112]. In turn, OxLDL has recently been reported to predict the development of renal dysfunction [113]. Another marker of oxidative stress in inflammatory diseases is 3-nitrotyrosine (TyrNO_2_) associated with the overproduction of NO [114].

Kidney disease is associated with permanent inflammation accompanied by oxidative stress [115]. Markers of inflammation include C-reactive protein, interleukins (IL-1, IL-6), tumor necrosis factor alpha (TNF-*α*), and fibrinogen, among others. Another marker is MPO, which has been found in serum and is associated with inflammation in CKD patients [116].

**Figure 3:**
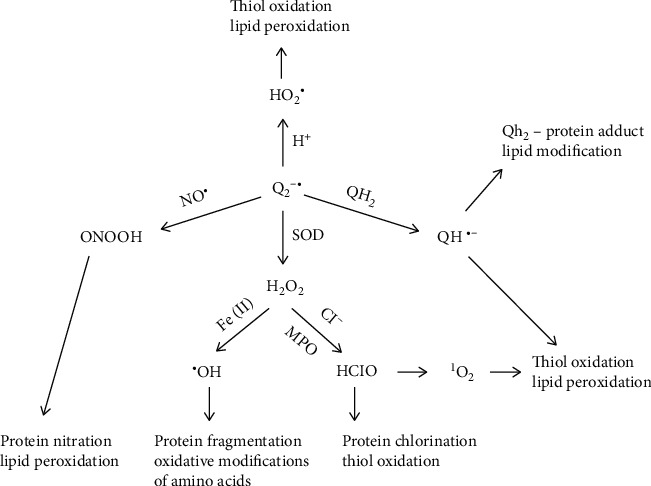
Reactive oxygen species production from superoxide anion radical and biological material damage by ROS. O_2_^•-^, a precursor of other ROS, such as H_2_O_2_, which can oxidize chloride to HClO in the presence of MPO, ^1^O_2_ from HClO, and NO which produce ONOO^−^ and HO^•^ release in the Fenton reaction.

On the one hand, there is an overproduction of ROS; on the other hand, the activity of antioxidant enzymes is reduced and the level of antioxidants with low molecular weight is lowered. The decrease in the activity of SOD, decrease in the level of GSH, and higher GSSG/GSH ratio were described in RBC from hemodialyzed patients [119, 121, 123]. Additionally, chronic kidney disease is associated with inflammation and sometimes acute infections. Contact of dialysis membranes with blood causes the activation of phagocytic cells, which in turn leads to respiratory burst in 15-20 min of hemodialysis [3]. A respiratory burst is characterized by a decrease in the level of neutrophils by about 80% and of monocytes by about 60% and the release of large amounts of ROS. Neutrophils belong to cells that are crucial in the innate immune response against pathogens. These cells, which release superoxide (O_2_^•-^) and hydrogen peroxide (H_2_O_2_), can be generated with xanthine oxidase [124] and NO^•^ by neutrophilic nitric oxide synthase (NOS) [125]. Nitric oxide synthesized by NOS affects various physiological functions but is also involved in pathology. Nitric oxide is characterized by low reactivity; as an inert molecule, it easily penetrates plasma membranes. However, in the presence of oxygen, it forms highly toxic nitrogen dioxide (NO_2_^•^), a strong oxidizing and nitrating agent [126]. Neutrophils are cells that actively participate in inflammatory and cardiovascular diseases. Neutrophils can also produce a strong oxidant peroxynitrite in the reaction of superoxide and nitric oxide [127]. Increased production of O_2_^•-^ and H_2_O_2_, catalyzed by xanthine oxidase, is accompanied by an increase in the synthesis of peroxynitrite, the factor responsible for damaging the biological material (Figure 3) [126, 128]. Moreover, neutrophils produce hypochlorous acid in the presence of myeloperoxidase located in neutrophil granules, where hydrogen peroxide oxidizes chlorides to HClO, a strong oxidizing and bacteria killing agent. Using appropriate antibodies, it was shown that hypochlorous acid was responsible for inducing atherosclerotic lesion damage to macromolecules [129, 130]. Inflammation contributes to oxidative changes in proteins caused by reactive oxygen species. ROS and modified proteins may contribute to the development of changes in blood vessels that lead to atherosclerosis. In addition to the development of atherosclerosis, oxidation of low-density lipoprotein (LDL) also leads to glomerular sclerosis. It has been shown that HClO can be an important modifier of proteins and lipids and is involved in atherosclerotic changes and inflammation [130]. Using spin trap DMPO (5,5-dimethyl-1-pyrroline N-oxide) in EPR spectroscopy, hydroxyl radical generation in the blood at 20 min of hemodialysis of CKD patients was found [131]. Additionally, using another spin trap, N-tert-butyl-alpha phenylnitrone and also DMPO superoxide anion radical production during hemodialysis were detected [132]. These conditions further increase the release of ROS and the associated damage to the biological material of the host. This is the case with CKD patients. Additionally, oxidative stress is increased due to the presence of uremic toxins. Moreover, these patients experience oxidative stress during hemodialysis, as the contact of blood with artificial dialysis membranes leads to a respiratory burst of neutrophils and the associated release of high amounts of ROS including oxygen free radicals [3]. Oxidative stress appears to be the leading cause of mortality in patients with chronic kidney disease (CKD) due to the high risk of cardiovascular disease.

As a result of oxidative stress, oxidation of amino acid side chains, oxidation of peptide backbones, cross-linking of proteins, and advanced oxidation protein products (AOPP), carbonyl compounds are also released and a decrease in plasma thiol group concentration was observed [133, 134]. Free thiol groups (-SH) are of key importance in protection against oxidative stress because they are very sensitive to oxidation by ROS *in vivo* [135]. Using the spin labelling technique in EPR spectroscopy, we showed changes in the structure of hemoglobin HbA1c and HbA in patients with CKD both before and after hemodialysis. Conformational changes also concerned the pool of nonheme proteins present inside the erythrocytes [136]. These changes were caused by oxidative stress. It was also shown that mild oxidative stress caused hemoglobin to bind to the plasma membrane [137]. Hemoglobin conformational changes in CKD patients were accompanied by a decrease in total thiols in hemolysate before and after hemodialysis. The conducted studies showed the loss of the -SH groups in HbA1c and HbA hemoglobin as well as in nonheme proteins [136].

The disturbance of the redox balance is associated with the increase in ROS production and a decrease in antioxidant capacity. In turn, irreversible oxidation of the residue of the cysteine *β*Cys93 in the globin chain may lead to disintegration of the structure of Hb and, consequently, to the release of heme, which also catalyzes free radical reactions [138]. There is a decrease in the activity of antioxidant enzymes [119, 121, 123]. Moreover, the amount of superoxide can be increased by activating the NADPH oxidase. Generally, it leads to oxidative stress, which causes disturbances in the structure and functioning of these cells, disintegration of the membrane, changes in its permeability, and hemoglobin leakage. The release of Hb from red blood cells can damage proteins, lipids, and other important molecules and macromolecules. Oxidative stress in CKD patients leads to the peroxidation of lipids and proteins. In the case of lipids, the end products of oxidation are malondialdehyde (MDA), isoprostanes, oxysterols, and 4-hydroxynonenal (HNE). For example, oxysterols initiate and worsen atherosclerosis [139]. In addition to lipid peroxidation, ROS leads to the oxidation of proteins, carbohydrates, glycoproteins, and others, of which the products are advanced glycation end products (AGE), carbonyls, and advanced protein oxidation products (AOPP). These products are also biologically active [140].

It has been shown that after hemodialysis, a decrease in the total antioxidant capacity and glutathione (GSH) in the blood was found, while a much higher level of MDA was noted. In addition, a decrease in the activity of glutathione peroxidase and superoxide dismutase was observed in erythrocytes before and after hemodialysis, while the activity of catalase increased [141]. The range of changes of these parameters was influenced by the dialysis membrane. Polysulfone membranes were characterized by greater biocompatibility than cellulose membranes, and the observed decreases in antioxidants were lower than those for cellulose membranes. Also, a smaller increase in the level of MDA was recorded for the polysulfone membrane than for the cellulose dialyzer [141].

Advanced glycation end products (AGEs) are produced in patients with chronic kidney disease [133]. High levels of these substances are due to decreased renal clearance. AGEs are produced in the Maillard reaction, a series of chemical reactions that occur between amino acids, lipids, nucleic acids, and reducing sugars. AGEs are also produced in diseases with high levels of oxidative stress. Interactions between AGEs and their receptors (RAGE- (receptor for advanced glycation end products-) transmembrane, immunoglobulin-like receptor) can initiate oxidative stress and inflammation, leading to cardiovascular complications. It has been shown that neutrophils can generate more ROS by responding to AGEs via the NADPH oxidase complex [142]. Additionally, ample evidence suggests that the interaction between AGE and RAGE has a significant effect on inducing vascular damage, including endothelial dysfunction and arterial stiffness [143].

## 5. Red Blood Cells in CKD

**Figure 4:**
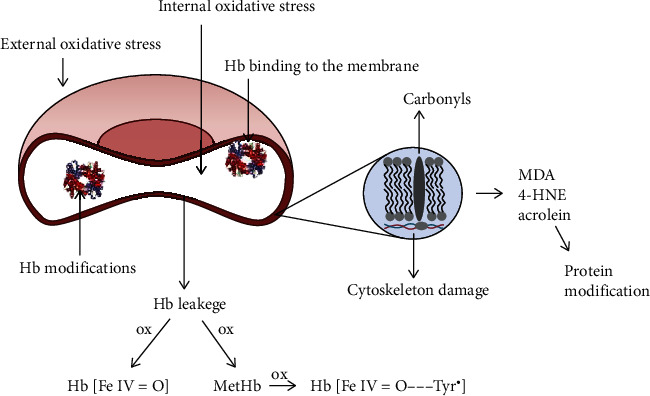
Red blood cell membrane damage from internal and external sources and hemoglobin (Hb) release. Ferryl (Hb[FeIV=O]) and ferryl radical form of hemoglobin (Hb[FeIV=O‧‧‧Tyr^•^]). MDA: malondialdehyde; 4-HNE: 4-hydroxynonenal.

Red blood cells (RBC) are permanently exposed to high oxygen concentration, which promotes the production of ROS. Within 24 hours, 3% of hemoglobin is oxidized and a superoxide radical is generated. In addition, hemoglobin itself is a catalyst for free radical reactions. Redox balance is maintained due to the presence of antioxidant enzymes and reducing agents with low molecular weight (Figure 4). Oxidative changes in erythrocyte components may lead to their deformation, which is influenced by the fluidity of the plasma membrane and the internal viscosity of RBC. In turn, the deformability of red blood cells is of key importance in microcirculation, because their diameter is larger than the diameter of the capillaries through which they flow. Oxidative damage to the RBC membrane has been reported to have a significant effect on the viscoelastic properties of the membrane [144]. In addition, the fluidity of the membrane is also important in the function of the RBC and the removal of toxic metabolites from the cell. Oxidative damage to the erythrocyte plasma membrane leads to impaired oxygen supply and leads to accelerated aging of red blood cells [145].

The red blood cells of patients with CKD had a greater fluidity of plasma membranes measured at different depths of the lipid monolayer than the RBC of healthy volunteers. The fluidity of the membranes increased with the time of hemodialysis. In the conducted experiment, we showed that RBCs from CKD patients were significantly more sensitive to oxidative stress induced by hydrogen peroxide than erythrocytes from healthy subjects [146]. The increase in membrane fluidity was accompanied by deepening changes in the membrane cytoskeleton [132, 146]. Moreover, plasma membranes of CKD erythrocytes were characterized by higher osmotic fragility compared to RBC of healthy individuals (Figure 2). Additionally, the fragility increased significantly after treatment with hydrogen peroxide [146]. It can, therefore, be assumed that each subsequent hemodialysis treatment can deepen the oxidative damage in these cells. Oxidative stress in the red blood cell leads to aging erythrocytes and phosphatidylserine exposure. The lifespan of a normal erythrocyte is 120 days. On the other hand, the lifetime of erythrocytes in chronic kidney disease is much shorter by up to 70% [147, 148]. Toxic uremic environment such as uremic toxins and oxidative stress shorten the survival time of red blood cells, which leads to anemia in chronic kidney disease. In the blood of patients, younger blood cells dominate, which are more susceptible to oxidative stress, and that may additionally contribute to the shortening of the survival time of young erythrocytes in patients with CKD.

There is a decrease in the activity of antioxidant enzymes [119, 121, 123]. In addition, the superoxide pool can be increased by activation of NADPH oxidase. In general, such a situation leads to oxidative stress, which causes disturbances in the structure and function of these cells. As a result of the oxidation of the components of the cell membrane, i.e. proteins and lipids, changes in its permeability occur, which leads to hemoglobin leakage.

Red blood cells can be damaged from both internal and external sources. The dominant factor of oxidative stress within the RBC is Hb. Oxygen derivative free radicals are generated as a result of autooxidation of Hb associated with the inner surface of the membrane, mainly with cytoskeleton proteins [149]. The released superoxide anion and the product of its dismutation, hydrogen peroxide, lead to the formation of hemichromes and degradation of heme, releasing free iron that catalyzes the Fenton and Haber-Weiss reactions. Additionally, hydrogen peroxide oxidizes the corresponding Hb and Met Hb to the ferryl form and the radical ferryl form.

Nitrite ions can diffuse into the interior of the erythrocyte, which are the source of NO or NO coming from the endothelial cells. However, most of the NO that the RBC is exposed to originate from endothelial e-NOS [150]. In turn, RBC can release the superoxide anion radical out through band 3. Thus, NO can also react with the superoxide in the cell and plasma to produce peroxynitrite, a powerful oxidant that can damage RBC inside and out [149].

Hemoglobin released from erythrocytes is dangerous because it is toxic and may initiate oxidation reactions in the biological material. To prevent damage to proteins, the lipid and other molecules are Hb bound by haptoglobin (Hp), which is an acute-phase protein that reduces oxidative damage. However, binding of haptoglobin to hemoglobin increases the level of ferryl formation during Hb-catalyzed lipid peroxidation. The increased stability of the Hp-Hb complex was also observed in the absence of lipids with the presence of external reducing agents [38]. The release of free Hb from red blood cells occurs in hemodialysis as a result of mechanical damage to red blood cells by dialysis pumps [151, 152]. We have repeatedly observed the presence of hemoglobin in the plasma of patients who have completed hemodialysis.

The free Hb released in plasma is bound by haptoglobin; however, with a high degree of intravascular hemolysis, the level of haptoglobin is too low for all of the released Hb to be bound. Hb dimers are then filtered by the glomeruli and reabsorbed through the proximal tubule. When the reabsorption capacity is exceeded, hemoglobin appears in the urine [153]. Both hemoglobin and myoglobin are prooxidants; if the released heme is not bound by hemopexin, then the iron redox cycle in the heme leads to globin radicals that induce lipid peroxidation [39]. Kidney damage by free Hb is similar to that by Mb in rhabdomyolysis, where Mb accumulates in the renal tubules, heme is released, and oxidative stress damages the renal parenchyma.

The products of lipid peroxidation are malondialdehyde (MDA) and 4-hydroxynonenal (4-HNE). MDA is more mutagenic compared to 4-HNE, which in turn is the most toxic product of lipid peroxidation [154]. Its high toxicity is caused by reactions with thiols and amino groups, which lead to stable adduct proteins [155]. 4-HNE is also a signaling molecule and second only to the lipid peroxide toxic messengers of free radicals. 4-HNE not only is a signaling molecule but also is involved in the regulation of several transcription factors such as factor 2-bound nuclear factor 2 (Nrf2), activating protein-1 (AP-1), NF-*κ*B, and receptors activated by peroxisome proliferators (PPARs). Furthermore, it performs roles in cell proliferation and/or differentiation, cell survival, autophagy, aging, apoptosis, and necrosis [156].

In chronic renal failure, many oxidized lipids have a toxic effect on cells and tissues, having a proapoptotic and proinflammatory effect, especially in the cardiovascular system. They include isoprostanes, especially isoprostane F2, of which the concentration increases with the development of the disease. Their accumulation and harmful effects meant that they were classified as uremic toxins. In CKD, oxidatively damaged lipoproteins are also observed, which lead to impaired HDL activity and may be strongly involved in accelerated atherosclerosis in patients with end-stage renal disease [157]. The risk of death in CKD patients is high due to cardiovascular complications [158]. Undoubtedly, premature death cannot be explained solely by the classic cardiovascular risk factors, such as hypertension, diabetes, and obesity. Recent studies show that the cause is uremic toxins, which are responsible for the increase in cardiovascular mortality in patients with CKD [159]. One of the toxins is spermine, a tetramine, but recent research has challenged this. It turned out that acrolein is the product of spermine oxidation with the participation of amine oxidase serum. Increased activity of serum amine oxidase leading to increased degradation of spermine was observed in CKD patients [160]. Acrolein is a very strong lacrimator, causing severe irritation to mucous membranes, eyes, and the upper respiratory tract. Already at a concentration of 2 ppm in the air, it can cause death. It was used for a period of time during World War I as a war gas. Acrolein is also released during lipid peroxidation, a process that is intensified in CKD patients.

## 6. Role of RBC in Cardiovascular Disease

**Figure 5:**
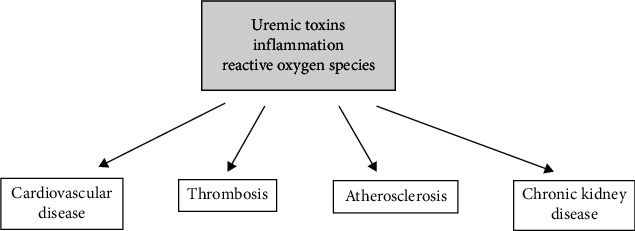
Uremic toxins, inflammation, and reactive oxygen species lead to chronic kidney disease, cardiovascular disease, thrombosis, and atherosclerosis.

The first cause of death in patients with end-stage renal disease on hemodialysis is a cardiovascular disease (CVD), which occurs in most patients. Mortality in this group is 20 times higher than that in the general population [161]. The short lifetime of RBCs in CKD causes anemia, a pathological condition characterized by a reduced number of circulating RBCs and a consequent low blood hemoglobin concentration compared to normal. Anemia can lead to serious complications of a cardiovascular disease (CVD), such as venous thrombosis, which can lead to stroke [162]. To increase the number of circulating erythrocytes, blood is transfused or erythropoietin is administered, which stimulates erythropoiesis. However, these actions do not always bring the expected results. Anemia leads to an increase in morbidity and mortality in cardiovascular diseases, which is associated with hypoxia. The consequence of hypoxia is an increased likelihood of thromboembolism, but also a hyperdynamic state associated with increased cardiac output, left ventricular hypertrophy and progressive enlargement of the heart, and possibly a proatherogenic role (Figure 5) [163].

The elderly are much more likely to develop venous thrombosis (VT)/thromboembolism (VT/E) due to the aging process characterized by an overproduction of reactive oxygen species (ROS). Red blood cells may perform a key role in initiating venous thrombosis during aging, according to recent studies (Figure 5). During RBC aging, RBC redox homeostasis is generally disrupted due to the imbalance between ROS production and the performance of antioxidant systems [164]. The main source of ROS is the autoxidation of hemoglobin and the activation of NADPH oxidase. RBCs can also be damaged by ROS from external sources and by other cells in the circulation. It has recently been shown that certain molecules produced during the blood clotting process can stimulate PMNs to produce ROS. ROS released by PMNs may damage RBCs, endothelium, and platelets and affect the coagulation process [165]. The consequence of the overproduction of ROS is oxidative damage to proteins and membrane lipids, which leads to a loss of membrane integrity and reduced deformability. These changes disrupt RBC functions in hemostasis and thrombosis, leading to hypercoagulability through increased RBC aggregation and RBC binding to endothelial cells, which may limit the availability of nitric oxide. In addition, RBC can activate platelets, modulating their activity. An important factor in hematology is the coagulation system and the activation of platelets, which not only contributes to hemostasis but also accelerates the coagulation system [166]. The interactions of RBCs with coagulation factors by influencing and activating them are also important.

During aging, the amount of ROS released increases, which disturbs the balance between thrombosis and hemorrhage. Emerging pathophysiological changes include disturbances in blood coagulation and related vascular function, blood flow, and coagulation pathways [167, 168]. Venous thrombus is characterized by a high content of RBC and fibrin; therefore, it is believed that red blood cells (RBCs) are now a critical mediator of venous thrombosis. Tissue factor (TF) is involved in the clotting pathway in the clotting process. Recently, the presence of TF has been demonstrated in neutrophils. It turned out that the interaction of neutrophils with endothelial cells is a critical stage, taking place earlier than the accumulation of platelets in the initiation of arterial thrombosis in damaged vessels [169].

Although RBCs are the major cellular component of venous clots, they are not the primary active causes of DVT. However, they influence the formation of blood clots through oxidative mechanisms. RBCs contain a large amount of hemoglobin which can autooxidize with the release of superoxide to form MetHb. Hb and MetHb can be, respectively, oxidized to the ferryl form and/or the radical ferryl form. Both forms initiate oxidative stress. The release of larger amounts of Hb from RBCs, which in the later stages intensifies the oxidative stress, leads to the activation of blood platelets, endothelial cells, and the formation of a thrombus [170]. These changes can be counteracted by haemoxygenase-1. HO-1 is an enzyme that catalyzes heme degradation and performs a key role in defending the body against oxidant-induced damage in inflammation. The role of HO-1 in the protection of the renal tubules against oxidative damage has been demonstrated. This enzyme is important as these cells are constantly exposed to oxidative stress. In an HO-1 deficiency, the renal tubular epithelium is more prone to oxidative stress [171]. Moreover, hemoglobin also increases the expression of functional TF in macrophages and reduces the sensitivity of TF to antioxidants [172]. Free hemoglobin generated during RBC hemolysis as a result of degradation releases heme that may initiate NETosis [173]. It has been shown *in vivo* that hemolysis associated with heme release activates inflammasome 3 (NLRP3) in macrophages. Macrophages, inflammasome, and IL-1R components have also been shown to contribute to hemolysis-induced mortality [174].

Systemic hypoxia has been shown to accelerate thromboembolic events through an inflammasome complex containing 3 (NLRP3) and increased IL-1*β* secretion. NLRP3 has also been shown to be mediated by inducible factor 1-alpha hypoxia (HIF-1*α*) [175]. It can be assumed that the above-mentioned factors associated with the pathology of venous thrombosis are more severe in patients with chronic kidney disease. This would partly explain the high mortality of CKD patients from cardiovascular diseases.

## 7. Conclusion

ROS are involved in inducing oxidative stress in the blood of patients and oxidative damage to the kidneys and in the development of chronic kidney disease. ROS are responsible for RBC damage, anemia and, consequently, general hypoxia of the body. Overgeneration of ROS leads to stimulation of the blood coagulation, activation of platelets, endothelial cells, neutrophils, and other processes that promote the formation of venous clots leading to thromboembolism and associated complications.

## Figures and Tables

**Table 1:** Markers of oxidative stress determined in saliva, plasma, and red blood cells in CKD.

Patients	Marker	Increase ↑/decrease ↓	Reference
Saliva (NWS)			
CRF (not requiring dialysis) vs. ESRD (peritoneal dialysis)	UA	↓	[117]
	TAS	↓	
	GPx	↑	
	SOD	↑	
Pediatric patients with CKD vs. healthy controls	UA	↑	[118]
	GSH	↓	
	CAT	—	
	GPx	—	
	SOD	↑	
	TAS	—	
	AGE	↑	
	AOPP	↑	
	MDA	↑	
Plasma			
CRF (not requiring dialysis) vs. ESRD (peritoneal dialysis)	UA	↓	[117]
	TAS	↑	
Pediatric patients with CKD vs. healthy controls	UA	↑	[118]
	GSH	↓	
	CAT	—	
	GPx	—	
	SOD	↑	
	TAS	↑	
	AGE	↑	
	AOPP	↑	
	MDA	↑	
CRF (treated by hemodialysis) vs. healthy controls	GPx	↓	[119]
	GR	↓	
	TBARS	↑	
	AOPP	↑	
	Carbonyl	↑	
CRF (treated by hemodialysis) vs. healthy controls	TAS	↑	[120]
Red blood cells			
ESRD (treated by hemodialysis) vs. healthy controls	SOD	↓	[121]
	CAT	↑	
	GPx	—	
CRF (treated by hemodialysis) vs. healthy controls	SOD	↓	[119]
	GSH	↓	
	GPx	—	
ESRD (treated by hemodialysis) vs. healthy controls	SOD	↓	[122]
	CAT	↓	
	GPx	↓	
	TBARS	↑	

NWS: nonstimulated saliva; AGE: advanced glycation end products; AOPP: advanced oxidation protein products; carbonyl: carbonyl group; CAT: catalase; GPx: glutathione peroxidase; GR: glutathione reductase; GSH: reduced glutathione; MDA: malondialdehyde; SOD: superoxide dismutase; TAS: total oxidant status; TBARS: thiobarbituric acid reactive substances; UA: uric acid.
